# Supplementary figures and images for: Specification of the endocrine primordia controlling insect moulting and metamorphosis by the JAK/STAT signalling pathway
Source: PLoS Genet. 2022 Oct 3;18(10):e1010427. doi: 10.1371/journal.pgen.1010427 (PMC9560620; doi:10.1371/journal.pgen.1010427)

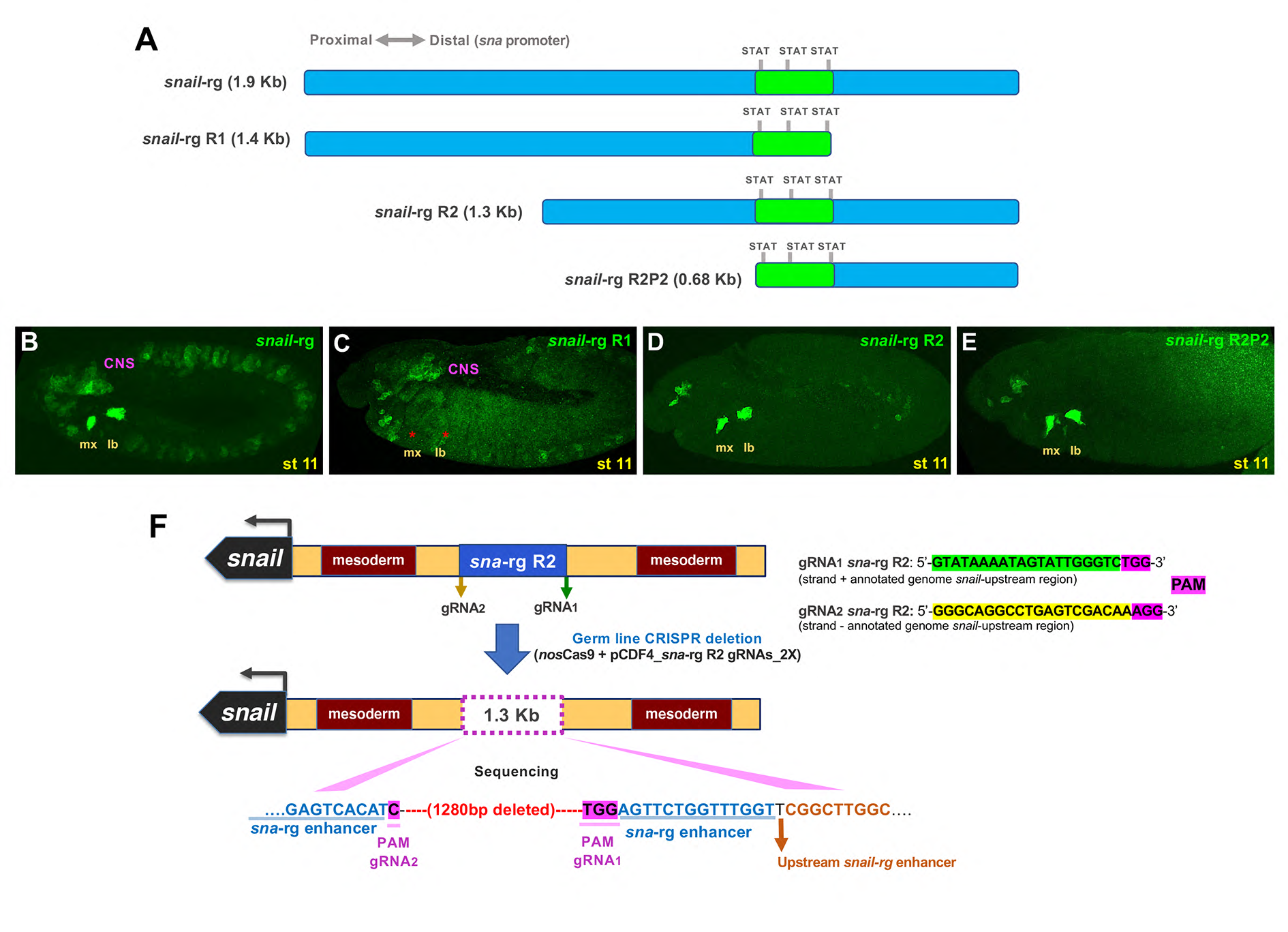

Supplement: S1 Fig — (A) Scheme of DNA elements tested indicating the location of the STAT binding sites. The sna-rg R2 reporter comprises the cis-regulatory sequence deleted in the CRISPR-Cas9 snaΔrgR2 mutation. Expression at st11 of the full sna-rg-GFP (B), sna-rg R1-GFP (C), sna-rg R2-GFP (D), and sna-rg R2P2-GFP (E) constructs. (F) Scheme showing the fragment deleted in the snaΔrgR2 mutation indicating the sequence of the sgRNAs used and the sequences flanking the deletion. (TIF) [file pgen.1010427.s001.tif]

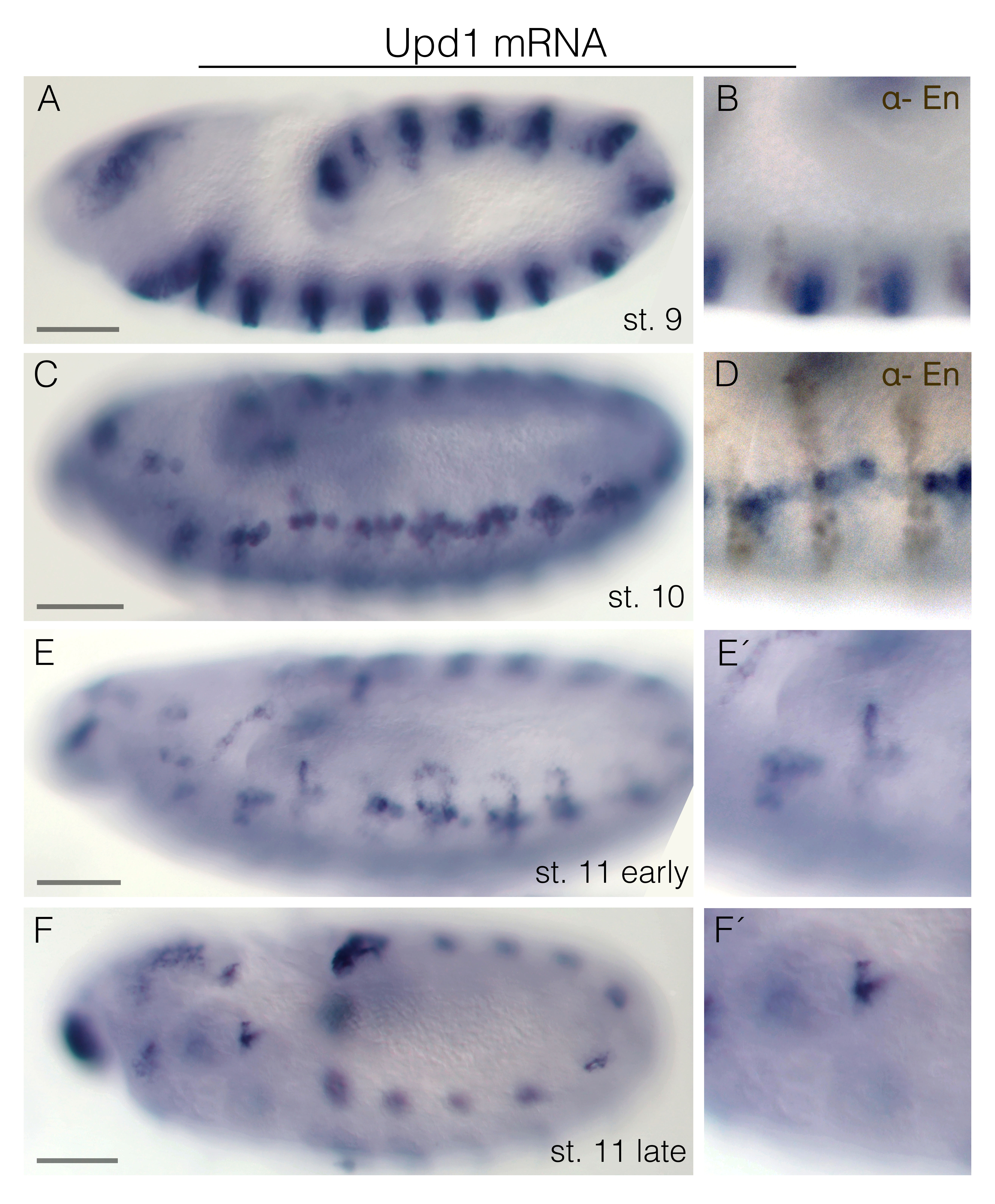

Supplement: S2 Fig — Whole mount in situ RNA expression in 5-7h wild type embryos. Right panels show close ups of the maxillary and labial segments. (A) At st9 upd is expressed in stripes, located posterior to the En stripe (B). (C) At st10 there is a transient upd anteroposterior stripe running along the lateral ectoderm. (D) Close up of a st10 embryo focusing at the maxillary and labial segments. (E-E’) At early st11 lateral ectoderm expression is detected in the gland primordia and in the tracheal pits. (F-F’) At late st11 upd expression disappears from the CA and is detected in the PG primordium and in the invaginating trachea. Panels (B-D) show embryos double stained with anti-En (brown). Scale bars 50 μm. (TIF) [file pgen.1010427.s002.tif]

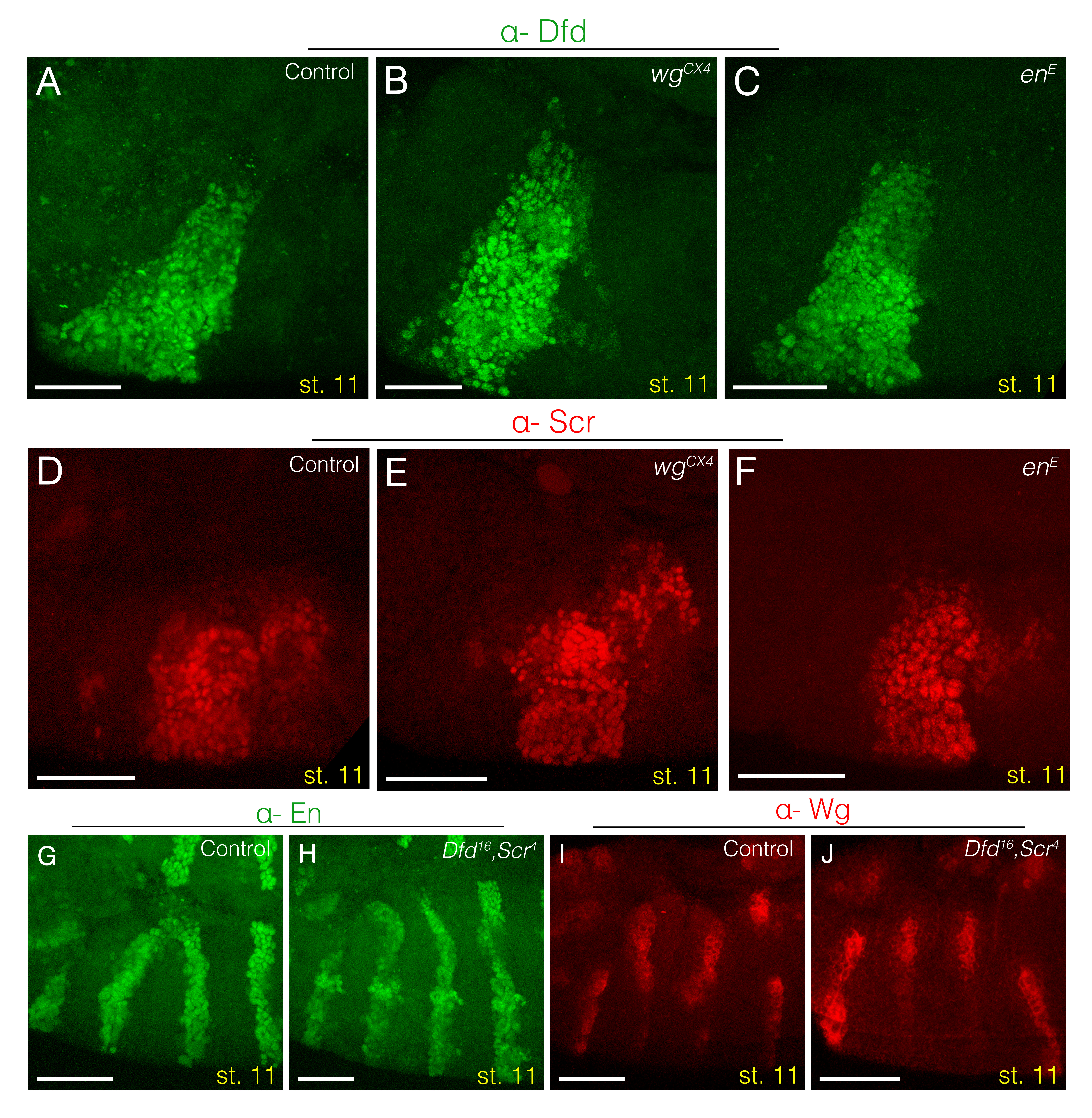

Supplement: S3 Fig — Dfd expression in (A) control heterozygous or in (B) wgCX4 and (C) enE homozygous embryos. Scr expression in (D) control heterozygous or in (E) wgCX4 and (F) enE homozygous embryos. En expression in (G) control heterozygous or (H) Dfd16 Scr4 embryos. Wg expression in (I) control heterozygous or (J) Dfd16 Scr4 embryos. Images show lateral views of the mandibular to the T1 segment in st11 embryos. Scale bars 50 μm. (TIF) [file pgen.1010427.s003.tif]

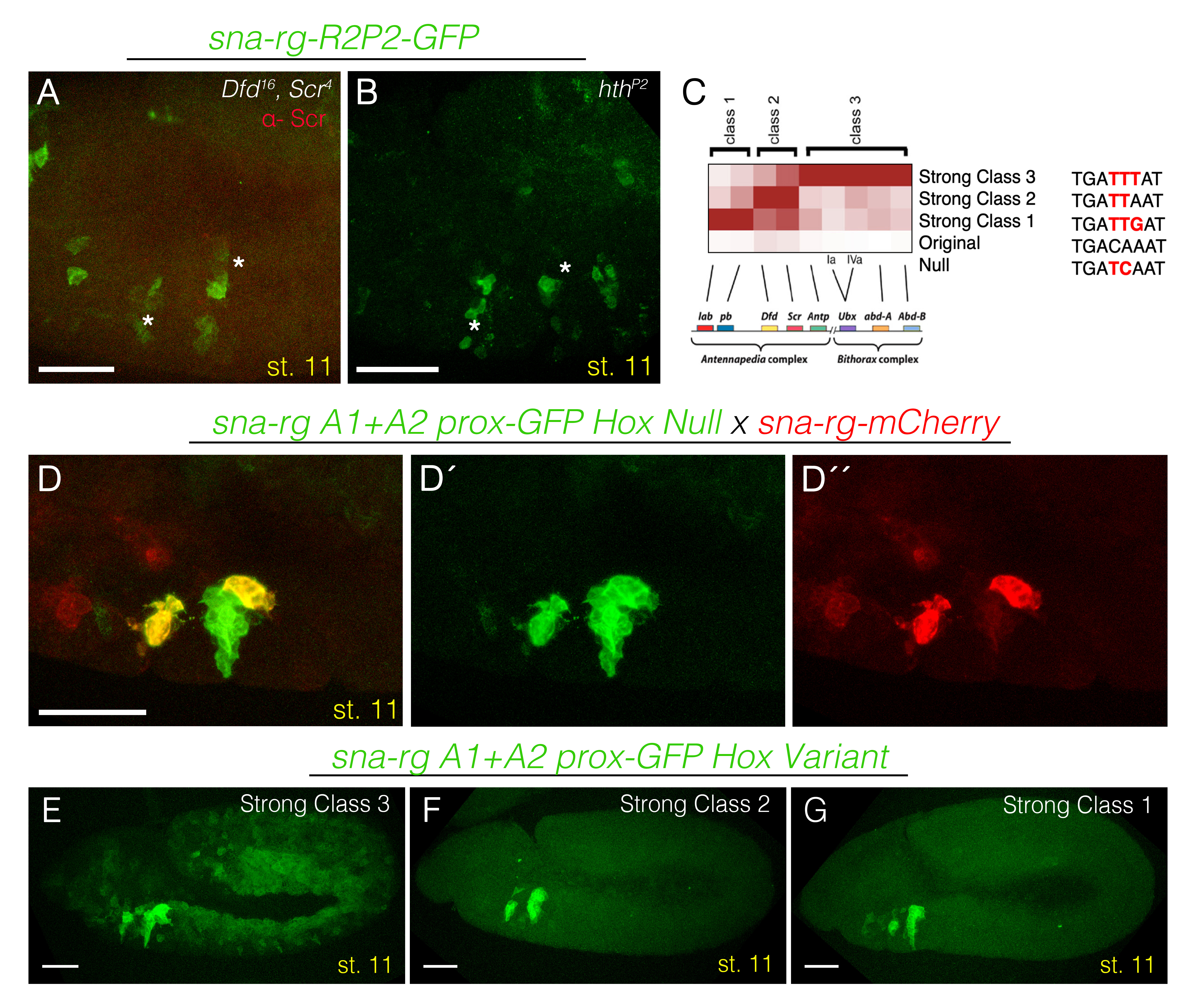

Supplement: S4 Fig — Expression of sna-rg-R2P2 minimal enhancer in a Dfd Scr double mutant (A) or in a hthP2 mutant (B). (C) Hox binding site modifications introduced in the sna-rg A1+A2proximal constructs beside a heat map indicating their SELEX-seq DNA-binding affinity preferences. (D) Embryo expressing simultaneously a sna-rg-mCherry construct (D” red) and sna-rg A1+A2proxHoxNull-GFP (D’ green) where the putative DNA binding site has been changed to a sequence not recognised by Hox-Exd-Hth in vitro. The expression of the sna-rg A1+A2proxHoxNull-GFP construct in the ring gland is not affected by the putative binding site mutation as well as maintaining the ectopic ventral expansion normal to a sna-rg A1+A2prox reporter. (E) sna-rg A1+A2prox strong class3Hox-GFP. (F) sna-rg A1+A2prox strong class2Hox-GFP. (G) sna-rg A1+A2prox strong class1Hox-GFP. Mutant embryos in (E-G) show strong expression in the maxilla and labium and the expression in other segments is barely affected. Panel C is modified from [40]. Embryos in (A) are also stained with anti-Scr to recognise the homozygous mutants. Scale bars 50 μm. (TIF) [file pgen.1010427.s004.tif]
