# Supplementary material for: Specification of the endocrine primordia controlling insect moulting and metamorphosis by the JAK/STAT signalling pathway
Source: PLoS Genet. 2022 Oct 3;18(10):e1010427. doi: 10.1371/journal.pgen.1010427 (PMC9560620; doi:10.1371/journal.pgen.1010427)
Supplement: S1 Table — (DOCX) [file pgen.1010427.s005.docx]

| Primer | Sequence |
| --- | --- |
| sna-rg for (KpnI) | 5'-ATGGTACCACCAAACCAGAACTCCAGACC-3' |
| SnaR2P2B3rev | 5'-ATGGTACCCCCGATAACCTTCTCGGG-3' |
| SnaR2P2B4for | 5'-ATGGTACCAGAAGGTTATCGGGGATC-3' |
| sna-rg R2P2 rev (KpnI) | 5'-ATGGTACCCTTCCCAGAAAACTGTAAGGC-3' |
| SnaR2P2B4rev | 5'-ATGGTACCATCCCATCTAAATCATTTATAAGCG-3' |
| SnaR2P2B5for | 5'-GCGGTACCCGGTAATTGTTGTCGC-3' |
| StatS1-NotI-for | 5'-GGCCGCAATTTTCCCTGGAAAAATTACG-3' |
| StatS1-NotI-rev | 5'-GGCCCGTAATTTTTCCAGGGAAAATTGC-3' |
| StatS1mut-NotI-for | 5'-GGCCGCAATTCGACCTGCTCAAATTACG-3' |
| StatS1mut-NotI-rev | 5'-GGCCCGTAATTTGAGCAGGTCGAATTGC-3' |
| Sna mut1 for strong Cl3 | 5'-GTCAGACAGCAGTGATTTATGAAAGCGAAACCCCGATCG-3' |
| Sna mut1 rev strong Cl3 | 5'-GCCCCAAAGCGAAAGTATTTAGTGACGACAGACTGTGGAAC-3' |
| Sna mut2 for strong Cl2 | 5'-GTCAGACAGCAGTGATTAATGAAAGCGAAACCCCGATCG-3' |
| Sna mut2 rev strong Cl2 | 5'-GCCCCAAAGCGAAAGTAATTAGTGACGACAGACTGTGGAAC-3' |
| Sna mut3 for strong Cl1 | 5'-GTCAGACAGCAGTGATTGATGAAAGCGAAACCCCGATCG-3' |
| Sna mut3 rev strong Cl1 | 5'-GCCCCAAAGCGAAAGTAGTTAGTGACGACAGACTGTGGAAC-3' |
| Sna mutNull for | 5'-GTCAGACAGCAGTGATCAATGAAAGCGAAACCCCGATCG-3' |
| Sna mutNull rev | 5'-GCCCCAAAGCGAAAGTAACTAGTGACGACAGACTGTGGAAC-3' |
| Fwd *sna-rg* BamHI | 5’-GGATCCACCAAACCAGAACTCCAGACC-3’ |
| R2P1 Rvs OP STAT mut | 5’-GTCATTGGAGATCCCGCAGCAGTGGCAAACA-3’ |
| Fwd SD STAT OP mut | 5’-GCTGCGGGATCTCCAATGACATCTTCATTTCCACGGTA-3’ |
| Rvs *sna-*rg R2P2 STAT mut BamHI | 5’-GGATCCCGTCCCAGAGAACTGTAAGGCTCGAATCTCCGGAATCCGTGGCAAACC-3’ |
| Upper BamHI sna-Med(R1) | 5’-TTCGAAGGATCCCTGCGGGATTTCCAATGA-3’ |
| Lower BamHI sna-rg | 5'-AGTTTGGGATCCGCTTGGGTTTTTCGTTTTCAG-3' |
| FwdSeq snaCRISPR | 5’-GGGGACGGGGGATTGGGATTC-3’ |
| RvsSeq snaCRISPR | 5’-ACTACCGCATTTATGATCGACATCGGAA-3’ |
