# Supplementary material for: Specification of the endocrine primordia controlling insect moulting and metamorphosis by the JAK/STAT signalling pathway
Source: PLoS Genet. 2022 Oct 3;18(10):e1010427. doi: 10.1371/journal.pgen.1010427 (PMC9560620; doi:10.1371/journal.pgen.1010427)
Supplement: Attachment — Submitted filename: 126288_1_rebuttal_2423069_rfnw5n.docx [file pgen.1010427.s006.docx]

## Manuscript number: RC-2022-01421

**Corresponding author:** James, C-G Hombría

*[The “revision plan” should delineate the revisions that authors intend to carry out in response to the points raised by the referees. It also provides the authors with the opportunity to explain their view of the paper and of the referee reports.*

*The document is important for the editors of affiliate journals when they make a first decision on the transferred manuscript. It will also be useful to readers of the reprint and help them to obtain a balanced view of the paper.*

*If you wish to submit a full revision, please use our "*[*Full Revision*](https://reviewcommons.org/templates/full-revision.docx)*" template.* ***It is important to use the appropriate template to clearly inform the editors of your intentions.****]*

## 1. General Statements [optional]

*This section is optional. Insert here any general statements you wish to make about the goal of the study or about the reviews.*

Reviewers 1 and 2 are very positive about our manuscript, while reviewer 3 is surprisingly critical.

However, except for the first observation, most of reviewer 3´s comments are based on incorrect interpretations of our results.

We have integrated the useful comment into our revised version, and we will discuss in the following sections why reviewer 3’s remaining criticisms should be disregarded.

## 2. Description of the planned revisions

*Insert here a point-by-point reply that explains what revisions, additional experimentations and analyses are planned to address the points raised by the referees.*

In the revised manuscript we are here submitting, we have included most of the suggestions made by the reviewers.

The only experiment we have not yet finished is the following one:

Reviewer 3 doubts that the activation of *upd* occurs in the sna-rg primordium when saying: “*Likewise, the STAT10X-GFP staining does not overlap with the sna-rg-mCherry staining (I see red cells and I see green cells - there are no yellow cells). If activation of snail is through Upd activation of STAT signaling, we should see that the snail reporter expression is within the domain of STAT10X-GFP expression*.”

This criticism should have referred exclusively to the CA primordia but not to the PG primordia where co-expression of *sna-rg-mCherry* and *x10STAT-GFP* in panel 4B should be evident to the reviewer. We believe that the lack of clear co-expression of GFP and RFP in the CA is due to the fact that *upd* activation in the CA is extremely transient, leading to the loss of the x10STAT-GFP expression before the *sna-rg-mCherry* levels are robust enough in the maxilla.

To try to sort the CA co-expression problem, we are currently repeating the experiment but instead of analysing *sna-rg-mCherry* activation with the RFP antibody, we will do an *mcherry* RNA *in situ*. We hope that the *mcherry* transcript will be detectable earlier than the protein and the co-expression will also be evident in the CA.

## 3. Description of the revisions that have already been incorporated in the transferred manuscript

*Please insert a point-by-point reply describing the revisions that were already carried out and included in the transferred manuscript. If no revisions have been carried out yet, please leave this section empty.*

**Reviewer 1:**

Reviewer 1 has only minor suggestions and is satisfied that we prove convincingly our claims. The reviewer also finds our results reinforce our previously proposed hypothesis that the glands and the trachea evolved from common metamerically repeated ancient primordia.

We have introduced the following changes to the text to accommodate Reviewer’s 1 minor suggestions.

Main suggestion: *Insert a paragraph in discussion explaining the relevance of new insights to more basal insects that do not form a ring gland.*

We have introduced at the end of Discussion the following paragraph (marked in red):

“Our analysis of *snail* activation in the CA and PG shows that these glands and the trachea share similar upstream regulators, reinforcing the hypothesis that both diverged from an ancient segmentally repeated organ. In *Drosophila melanogaster* the CA and the PG primordia experiment a very active migration after which they fuse to the *corpora cardiaca* forming the ring gland (Sanchez-Higueras and Hombria, 2016). This differs from more basal insects where the CA fuses to the *corpora cardiaca* but not to the PG, and from the Crustacea where the three equivalent glands are independent of each other (Chang and O'Connor, 1977; Laufer et al., 1987; Nijhout, 1994; Wigglesworth, 1954). As the mechanisms we here describe relate to the early specification of the glandular primordia in *Drosophila*, it will be interesting to investigate if the equivalent genes are also involved in the endocrine gland specification of more distant arthropods”.

Additional comment 1: *Introduction, pg 3, a paragraph starting with "In comparison to the extensive knowledge we have of ..." - consider omitting or greatly shortening, this text breaks a flow as it is focused on tracheal development. I understand the authors' logic, but this information distracts from the main focus on CA and PG.*
We agree that the trachea description paragraph breaks the flow of the introduction to gland development. As suggested by the reviewer, we have deleted most of the descriptive text on trachea development but left all the references so that interested readers can find the information.

Additional comment 2: *Beginning of discussion, pg 11:
- change 2nd sentence to: " Our results indicate that the HH and the Wnt pathways act indirectly to negatively regulate the spatial activation ..."
- the following sentence, starting with "Engrailed activation off hh transcription ...." is way too long and hard to follow, consider breaking into two sentences.*
We have changed both sentences as suggested.

Additional comment 3: *In Fig 4B, mx and lb segments should be labeled so this panel is consistent with labeling in 4A.*
We have changed Fig.4B labels to be consistent with 4A

Additional comment 4: *In Fig 6, reduce a font size for labels on right-hand side (A1, A2, A1+A2 proximal, etc), so that they are visualy distinct from panel labels on left-hand side (A, B, C,..).*

We have changed Fig.6 Font size as suggested

**Reviewer 2**

The reviewer is positive and agrees that the results we present in “*this paper add to our understanding of how the CA and PG primordia are specified and highlights important similarities with the specification of the tracheal primordia*”. The reviewer’s comments focus specially on the activation vs. maintenance of *sna*.

Specific Comment a): Referring to Fig 1G-J, the reviewer says*: It is not clear to me from either this figure or from the text whether the initial pattern of expression of the sna-rg reporter in stage 11 embryos is WT and then disappears at stage 12, or whether it is always defective. In trying to understand the activation process, I think it would be important to know for sure whether rg enhancer activity during the initiation phase in stage 11 is WT or not.*

As suggested by the reviewer, we have included st11 embryos in Fig. 1 as panels G,J which illustrate that early *sna-rg* activation occurs normally in *sna^ΔrgR2^*embryos prior to apoptosis kicking in. To make space for these images, we have taken out the st12 embryos that we had in our previous submitted version. This does not affect the manuscript’s message, as st12 phenotypes are similar to those at st13 which are presented in Fig. 1H,J.

Moreover, in this revised version, the embryos in Fig. 1G-J have also been double stained with the apoptosis marker DCP1 to highlight the cell death observed in the gland primordia of *sna^ΔrgR2^* embryos (Fig. 1G’-J’).

Specific Comment b) See section 4 **“**Description of analyses that authors prefer not to carry out”

Specific Comment c) See section 4 **“**Description of analyses that authors prefer not to carry out”

Specific comment d) *The authors show that there is an expansion in the number of sna-rg reporter expressing cells along the AP axis when upd is ectopically expressed using a sal-Gal4 driver. Though not mentioned in the text at this juncture, sal is expressed in the PG primordia, while seven-up (svp) is expressed in the CA primordia. I assume that the upd induced expansion is only observed for the PG primorida (LB) and not the CA primordia (Mx)-at least this is what the figure looks like. (…) How about svp driven upd-assuming there is a svp-Gal4 driver-does it cause an expansion of Ca but not PG.*

As the reviewer has noticed, there is a stronger expansion of *sna-rg-GFP* expression in the labial segment than in the maxillary segment. This is not due to the use of the *sal-Gal4* line. We see the same effect with *arm-Gal4* which drives similar expression on the maxilla and the labium. To illustrate this point, we have included two new panels (Fig.5D-E) where the ectopic expression of Upd has been induced with *arm-Gal4.* These embryos have been stained with anti-Sal to label the PG. This experiment shows clearly that the PG has expanded much more than the CA.

There are several reasons why expansion of the glands could be more efficient in the labium than in the maxilla. One possible reason is the temporal response to Upd activation. Upd induction by the *arm-Gal4* and *sal-Gal4* lines may occur after the cells in the maxilla are no longer capable of activating *sna-rg* but still capable of activating it in the labium. This temporal hypothesis is based on our results showing that the CA expresses more transiently the *upd* gene and that STAT activation lasts for longer in the labium than in the maxilla (Fig. 4A-D)].

A second possibility, that we favour, is the existence of dorso-ventral repressor genes modulating *sna-rg* expression intrasegmentally. Some of our results point towards the *sna-rg* CRM receiving repressor inputs that modulate intrasegmental spatial expression in the dorso-vental axis. When we delete the A2 distal region of the *sna-rg* enhancer, its expression in the labium expands ventrally (Fig. 6E,G and Sup.Fig. 4D). If a similar repressor was also modulating *sna-rg* in the maxilla it could be blocking its expansion. However, at this stage we have no solid data to support any of these hypotheses. As explained before for the maintenance mechanisms of *sna-rg* expression, our ongoing work aims to isolate and characterize further elements controlling the ring gland gene network, including these negative regulators.

In the revised manuscript we now describe the different effects of Upd ectopic activation on the expression of *sna-rg* in the maxilla and the labium (underlined text is new to this revised version):

“To test if generalised Upd expression in the maxilla and labium can activate *sna-r*g expression independently of other upstream positive or negative inputs, we induced *UAS-upd* with either the *sal-Gal4* or the *arm-Gal4* lines. We observe that, these embryos have expanded *sna-r*g expression along the antero-posterior axis in the maxillary and labial segments (Fig. 5C). Analysis of Sal expression, which labels the PG primordium (Sanchez-Higueras et al., 2014), shows that Upd ectopic expression induces a moderate expansion of the CA primordia while resulting a much larger increase of the PG primordium (Fig. 5D-E). This expansion occurs mostly in the anterior and posterior axis from cells where the Hh and the Wnt pathways are normally blocking *sna-rg* expression, while expansion is less noticeable in the dorso-ventral axis. This indicates that most of the antero-posterior intrasegmental inputs provided by the segment polarity genes converge on Upd transcription but that the dorso-ventral information is registered downstream of Upd.”

The differential response of *sna-rg* to Upd activation in the maxillary and labial segments is also mentioned in Fig. 5 legend. (see Continuation comment d).

*Continuation comment* d) “*It looks to me also like the vvl domain is expanding as well. This information should be clarified.”*

Yes, ectopic *upd* expression also expands *vvl1+2* expression. We have previously published that *vvl1+2* is a direct target of JAK/STAT signalling in the trachea (Sanchez-Higueras 2019 and Sotillos et al. 2010 Dev.Biol). Although *vvl1+2* expands dorsally in the Mx, those cells do not activate *sna-rg* dorsally. The ventral restriction of *sna-rg* in the maxilla is controlled by Dfd while in the labium its dorsal expression depends on Scr. We explain this in Fig.5’s figure legend where we now say (underlined text is new to this revised version):

**(C)** Ectopic Upd expression driven with *sal-Gal4* induces ectopic *sna-rg* and *vvl1+2* expression in the gnathal segments, which for *sna-rg* is more pronounced in the labium than in the maxilla. Note that in the maxillary segment Upd can induce ectopic dorsal *vvl1+2* but not *sna-rg* expression, this is expected as Dfd only induces *sna-rg* ventrally in the maxilla. (D-E) *sna-rg-GFP* embryos stained with anti-GFP (green) and anti-Sal (red). In control embryos **(D)** Sal labels the PG primordium but not the CA. In *arm-Gal4* embryos ectopically expressing Upd, the PG is more expanded than the CA as shown by number of cells co-expressing Sal and GFP.

*Specific Comment e) The authors note a difference between CA and PG in the requirement for STAT binding sites in the enhancers. Is that related to the fact that svp is expressed in CA and sal is expressed in PG? Would driving svp expression using the sal-Gal4 driver maintain sna-rg expression.*

During our preliminary ongoing experiments on *sna* maintenance mechanisms we looked in *svp* mutants and did not notice a change in *sna-rg* expression, thus it is unlikely that Svp is responsible for the difference. As said above, we continue looking for genes involved in gland formation. Sal could be involved in the maintenance of *sna* in the PG, but as Sal is expressed in the maxilla and labial segments before gland formation, it is difficult to disentangle if Sal is required for *sna* activation or maintenance (or both).

Specific Comment f) *Do svp or sal have a role in initiating sna expression when upd is present or maintaining sna expression after upd disappears? Presumably there is already published data that would answer these questions.*

As explained above we did not find any effect of *svp* on activation of *sna-rg*, however we find that in *sal* mutants the labium does not express *sna-rg.* This shows that *sal* is likely to be another positive input. As in *sal* mutants both *trh* and *Ubx* become ectopically expressed in the Lb (Casanova1989 *Roux's archives of developmental biology* 198: 137-140; Castelli-Gair 1998 IJDB42:437-444) we have done the experiment in *sal trh* double mutants and in *sal Ubx,abdA,Abd-B* mutants. In both cases we still see a failure of *sna* activation in the Lb reinforcing the idea that Sal is an additional positive input. However, we prefer not to add the *sal* experiments as they would complicate the paper which currently focuses on the similar requirement of the Wnt, Hh and JAK/STAT signalling pathways.

**Reviewer 3**

Reviewer is very critical. We accept some of the points raised and have modified the manuscript accordingly. However, as we detail below, the most serious criticisms are incorrect and do not affect the conclusions reached by our work.

We agree with the following comment:

*“In the Dfd Scr double mutant, both the CA and PG expression of the snail-rg-GFP reporter is still there - admittedly, the gland cells look abnormal at late stages, but this reporter that is supposed to function as a proxy for gland induction is still expressed. That either means that expression of sna-rg-GFP is not a proxy or that the glands are still being specified in the absence of the Hox genes that are proposed to specify these organs. The reporter should not be expressed if these Hox genes are what specify these endocrine organs.”*

The reviewer has made a good observation. The expression of *sna-rg-GFP* is not completely absent in *Dfd Scr* mutant embryos (Fig. 5F in this revised version), which indicates that although the Hox genes are required to activate *upd* in the maxilla and labium and in their absence the gland primordia become apoptotic, there must be other positive inputs to the enhancer. However, this does not mean the Hox gene input is irrelevant for gland specification. Not only the Hox genes are required to keep normal levels of *upd* expression in the Mx and Lb primordia and gland viability, but previously we also showed that cephalic Hox genes influence the dorso-ventral position inside the *vvl1+2* expressing cells where the *sna-rg* enhancer is activated: in the maxilla Dfd induces the ventral *vvl1+2* expressing cells to activate *sna-rg,* while in the labium Scr induces the dorsal *vvl1+2* cells to activate *sna-rg* (Sanchez Higueras 2014). The data presented in this paper indicate that the input of both Dfd and Scr over *sna-rg* CRM activation are indirect.

As a result of the reviewer’s criticism, we have tested if the additional positive input could be provided by Ci. In our previous submitted version, we showed that the repressor form of Ci blocks *sna-rg* activation. In this revised version, we have tested what is the effect of expressing the activator form of Ci. In embryos overexpressing the activator Ci^PKA^ isoform, we have observed that the expression of *sna-rg* and *upd* are expanded, indicating that Ci can provide the additional Hox-independent positive input. In the revised version we present these new results as Fig.3G and Fig. 4I. We have modified accordingly the scheme that appears in panel 3I to include this. In the main text we describe the result in the Hh regulation section where we have added:

“Although the above results indicate Ci is not absolutely required for *sna-rg* expression, we observed that overexpression of Ci^PKA^, the active form of Ci, causes a non-fully penetrant expansion of *sna-rg* expression (Fig. 3G) suggesting the possibility that *sna-rg* may be responsive to Ci and to a second activator.”

… and in the “Regulation of Upd ligand expression by the Wg and Hh pathways” section

where we say:

“We also found that ectopic expression of the activator Ci protein results in a non-fully penetrant expansion of *upd* expression in stage 10 embryos (Fig. 4H-I).”

We have also modified the final scheme in Fig. 7 to mention that Dfd and Scr prevent the apoptosis of the gland primordia, and that there must be an additional positive input controlling *upd* activation besides the Hox input. However, in the figure we do not define Ci as the activating input as we would like to have additional evidence before making such claim.

To clarify that the Hox input is not absolutely required we have modified the text in several places. Where we said:

“Expression of the *sna-rg* reporter in the maxilla and the labium requires Dfd and Scr function …”

We now say:

“Development of the CA and PG and normal expression of the *sna-rg* reporter in the maxilla and the labium require Dfd and Scr function …”

We also mention this in Fig. 5 legend where we have added:

“In *Dfd Scr* mutant embryos (F), although the gland primordia become apoptotic, residual GFP expression indicates that there must exist Hox independent inputs activating the *sna-rg* enhancer.”

As a result of reviewer 3’s comment, we have noticed a further example of similarity between the gland and the trachea specification, which we have commented in the revised discussion where we added the following paragraph:

“Another interesting similarity between glands and trachea is that, although ectopic Hox gene expression can ectopically induce *sna-rg* and *trh* outside their normal domain, the lack of Hox expression does not completely abolish their endogenous expression, indicating that in both cases a second positive input can compensate for the absence of Hox mediated activation. Our results suggest that, in the glands, this redundant input could be provided by the activating Ci form (Figs. 3G and 4I), but further analysis to confirm this possibility and discard alternative *sna-rg* activators should be performed.”

We disagree with the following comments:

*The finding that the CA and PGs form in slightly different DV positions from each other and slightly different DV positions from the trachea (based on the vvl1+2 mCherry reporter staining combined with that of the sna-rg-GFP reporter staining in Figure 5A, where staining does not overlap except where the CA cells have started to migrate over the vvl1+2 mCherry expressing cells) argues pretty strongly against the CA and PG being homologous to each other or absolutely homologous to the trachea primordia*

This erroneous claim was based on Fig. 5A, that showed a double stained embryo where co-expression is difficult to appreciate without separating the channels. Co-expression of these two reporter lines in the ring gland has been previously documented beyond doubt in our 2014 publication, cited throughout the manuscript, where we presented eight different panels of glands clearly co-expressing both markers at various developmental stages (Current Biology 2014 Fig.2B-I). To prevent any readers reaching the same conclusion as the reviewer, we have modified Fig. 5A to show a double stained *sna-rg-GFP* *vvl1+2-mCherry* embryo alongside with the two separate channels (panels 5A’ and A’’) to make the co-expression evident.

Although we are not including it in this manuscript, the reviewer will also be able to find images in the same 2014 Current Biology publication (Fig.3), where the ectopic activation of Dfd in the trunk leads to the activation of the *sna-rg-GFP* reporter in the *vvl1+2* tracheal cells, proving that the glands and the trachea are formed at homologous positions.

Having made clear that *sna-rg* activation in both the CA and the PG occurs in *vvl1+2* expressing cells, we now refute a second criticism: The reviewer is puzzled that despite the glands being formed at different dorso-ventral positions in the *vvl1+2* expressing patch of cells, we claim both groups of cells are homologous to the trachea.

We are not saying that the CA are formed at homologous positions to those giving rise to the PG. What we say is that both the CA and the PG are formed at positions homologous to those giving rise to the trachea in the trunk segments.

To make this clear in the revised version, we have changed the wording of a sentence in the Introduction section that might have originated the confusion.

Instead of saying:

“First, the CA, the PG and the traqueal primordia are specified in the lateral ectoderm at homologous positions”.

Now, it reads:

“First, the CA and the PG are specified in the cephalic lateral ectoderm at homologous positions to those forming the tracheal primordia in more posterior trunk segments.”

It has been shown that each tracheal primordium (which are labelled by *vvl1+2-mCherry*) gives rise to different tracheal branches depending on the positions where they are specified: the dorsal cells give rise to the dorsal tracheal branches, the ventral cells to the ganglionic branches, the medial cells to the dorsal trunk etc. (for illustration see Fig.12 in Manning and Krasnow 1993). Each of these tracheal branches have a different shape and migrate to different positions. We believe that a similar positional specification occurs in the *vvl1+2* cells in the maxilla and the labium. In the maxilla only the *vvl1+2* ventral cells activate *sna* and *svp* (among other genes) to give rise to the CA. In the labium *vvl1+2* dorsal cells activate *sna*, *sal*, *phm* (among other genes) to give rise to the PG. This regionalization is similar to what happens during tracheal branch specification, with the only difference that the interaction with Dfd and with Scr is what makes the positional outcome in the maxilla and the labium different (see in our Current Biology 2014 publication Fig. 3E-F and H-J). Thus, when the reviewer considers the equivalence between the CA/PG/trachea homology with that of the wing/haltere or that of the thoracic leg1/2/3 saying: “*Indeed, the situation with these endocrine glands and the trachea is completely unlike the situation with the wing and haltere, wherein both structures arise from the same DV position in adjacent segments, or with legs 1, 2 and 3, which arise from the same DV position in adjacent segments”*

…the reviewer should think about the coxa and the tarsi in the legs. The coxa in T1 is not homologous to the tarsi in T2 or T3, but when considering the leg structure as a whole, the coxa and the tarsi form part of the same homologous structure in T1, T2 and T3 despite being formed at different positions inside the leg primordia.

The reviewer also doubts that the activation of *upd* occurs in the sna-rg primordium when saying: “*Likewise, the STAT10X-GFP staining does not overlap with the sna-rg-mCherry staining (I see red cells and I see green cells - there are no yellow cells). If activation of snail is through Upd activation of STAT signaling, we should see that the snail reporter expression is within the domain of STAT10X-GFP expression*.”

This is due to the fact that *upd* activation in the CA is extremely transient, leading to the loss of the x10STAT-GFP expression before the *sna-rg-mCherry* levels are robust enough in the maxilla. This criticism does not apply to the PG where due to *upd* expression lasting longer, co-expression of *sna-rg-mCherry* and *x10STAT-GFP* in panel 4B should be evident to the reviewer.

To try to sort the CA co-expression problem, we are currently repeating the experiment but instead of analysing *sna-rg-mCherry* activation with the RFP antibody, we will do an *mcherry* RNA *in situ*. We hope that the *mcherry* transcript will be detectable earlier than the protein and the co-expression will be evident.

We strongly disagree when the reviewer says: “*This paper provides a strong basis for arguing that the CA and PG are induced independently of Jak/Stat signaling, whereas trachea require this signaling pathway*.”

When making this claim, the reviewer is ignoring a large number of experiments presented in the manuscript. If the CA and the PG are induced independently of JAK/STAT signalling:

(1) Why *sna-rg* expression disappears from the glands in mutants lacking the Upd ligands (Fig. 5B and 6K)?

(2) Why deleting the region containing the putative STAT binding sites in the *sna-rg* enhancer causes the loss of enhancer expression (Fig. 6C)?

(3) Why the smaller enhancer mentioned in point (2) recovers gland expression when adding a STAT binding site from an unrelated gene (Fig. 6G)?

(4) Why the regained expression of the construct mentioned in (3) is lost by the mutation of two bases affecting this single STAT site (Fig. 6H)?

The reviewer’s conclusion rests on giving an excessive importance to his reservations to CA co-expression in panel 4A while, surprisingly, disregarding the co-expression in the PG shown in panel 4B and all the experiments presented in Fig. 5 and Fig.6.

*Reviewer 3 Minor comments:*
Both comments have been taken into account in the revised version.

In summary, in this revised version we have answered most queries raised by reviewers 1 and 2. Moreover, reviewers 1 and 2 agree that the results presented in this manuscript reinforce the hypothesis that the CA and the PG glands and the trachea derive from the divergent evolution of a metamerically repeated homologous organ.

Reviewer 3 has made a good point that we have taken into account and has improved the revised submission.

However, reviewer 3 is wrong when concluding:

“*This paper provides a strong basis for arguing that the CA, PG and trachea are not homologous structures”*, and when saying: *“the CA and PG are induced independently of Jak/Stat signaling, whereas trachea require this signaling pathway”.*

As we argue above, these conclusions are erroneous because:

(1) Are based on the incorrect interpretation of Fig 5A and ignore previous published evidence cited throughout the manuscript.

(2) It does not take into account key experiments presented in this work, while giving too much weigh to a result that can be easily interpreted.

(3) It misinterprets the arguments justifying the positional homology between the CA/PG glands and trachea primordia.

## 1. Description of analyses that authors prefer not to carry out

*Please include a point-by-point response explaining why some of the requested data or additional analyses might not be necessary or cannot be provided within the scope of a revision. This can be due to time or resource limitations or in case of disagreement about the necessity of such additional data given the scope of the study. Please leave empty if not applicable.*

**Reviewer 2’s** Specific Comment b) *The authors argue that the rg deletion removes the only region driving sna expression in CA/PG. I'm not convinced that necessity necessarily implies sufficiency with respect to the requirements for rescue. While the sna-rg reporter is expressed in a pattern that seems to mimic the endogenous gene, do we know that a rg-sna transgene would fully rescue the rg deletion mutant?*
In our previous paper (Sanchez-Higueras 2014) we presented evidence that in *sna* null embryos, a Snail BAC gene lacking the *sna-rg* CRM can fully rescue the mesoderm phenotypes but not the ring gland ones. This proved that in the BAC transgene there was no shadow CRM capable of rescuing the gland formation in the absence of *sna-rg*. In the current paper we show that deleting the endogenous *sna-rg* CRM in the *sna* locus results in the absence of *sna* transcription from the gland primordia.

Making a *sna-rg-* construct expressing *sna* to test if this rescues the *sna^ΔrgR2^* homozygous mutants could be done, but it will delay this publication without adding much to the paper: we already know that *sna-rg* is sufficient to drive activation in all the CA and the PG cells (Sanchez-Higueras 2014 Fig 2J-M) and it would be expected to rescue the gland formation in *sna^ΔrgR2^* homozygous mutants.

Having said that, we have changed the wording in the manuscript to one that may be acceptable to the reviewer.

Instead of:

“These results prove that *sna^ΔrgR2^* deletes the only regulatory region driving *sna* expression in the CA and PG gland primordia…”

We now say:

“These results prove that the *sna^ΔrgR2^* ~~deletes~~ mutation inactivates the only regulatory region driving *sna* expression in the CA and PG gland primordia…”

**Reviewer 2’s** Specific Comment c) *is Sna required for maintaining sna expression?*
This experiment is relevant to the maintenance mechanism of *sna* expression in the ring gland, and not to its activation which is the main focus of this paper.

The search for the maintenance mechanisms is currently been followed in the laboratory and we prefer not deal with it in this paper. Providing a negative answer to this question would not be satisfactory, as we would need to search for the factors controlling *sna*’s maintenance.
